# Spatiotemporal dynamics of rhizosphere microbial communities in alfalfa across saline-alkali agro-ecosystems

**DOI:** 10.3389/fpls.2026.1792882

**Published:** 2026-05-28

**Authors:** Zhang Yutong, Liu Yaling, Yan Wei, Shi Fengling

**Affiliations:** 1 Key Laboratory of Grassland Resources, Ministry of Education P.R. of China, College of Grassland, Inner Mongolia Agricultural University, Hohhot, China; 2 National Technology Innovation Center for Prataculture, Hohhot, China; 3 Inner Mongolia Academy of Science and Technology, Hohhot, China

**Keywords:** alfalfa, community, environmental factors, metagenomics, spatiotemporal response

## Abstract

The rhizosphere represents a highly active plant–soil interface, where microorganisms play critical roles in the growth and development of alfalfa and in regulating local ecosystem processes. However, the mechanisms by which alfalfa rhizosphere microorganisms respond to spatiotemporal variation in saline–alkali environments remain poorly understood. Here, we collected alfalfa plants from one- to eight-year-old stands across three pastoral regions differing in soil type and characterized their rhizosphere soils. Using soil physicochemical analyzes, soil enzymology, and metagenomics, we examined how rhizosphere microbial communities respond to temporal and spatial variation in saline–alkali soils. Our findings indicate that alfalfa rhizosphere microecology may maintain rhizosphere health by modulating soil physicochemical properties, reducing peroxidase activity, enhancing reductase activity, and increasing the abundance of beneficial microorganisms. These results underscore the potential value of introducing exogenous beneficial bacteria to shape indigenous rhizosphere microecology.

## 1 Introduction

Soil salinization is a major environmental constraint that severely impairs agricultural soil function in arid and desert regions worldwide. Currently, approximately 8.3 × 10^6^ km² of global land area is affected by natural salinization (Heng et al., 2021). Salinization markedly reduces crop growth and productivity and exacerbates groundwater salinity, especially in arid to semiarid climates, ultimately accelerating soil degradation (Sherif et al., 2019; Chen et al., 2021). Although soil microorganisms constitute less than 0.5% of total soil mass, they are among the most active ecosystem components, driving nutrient cycling and serving as essential indicators of soil quality and productivity (Yan et al., 2015; Zhang et al., 2017). Microorganisms regulate mineral nutrient turnover, organic matter formation, and litter decomposition; they also influence plant growth and the suppression of pests and diseases (Schmid et al., 2021).

In China’s Inner Mongolia grasslands, comprising 21.7% of the country’s total grassland area, 31.8% is characterized by naturally barren, highly weathered, saline–alkali soils (Yang et al., 2018; Wang et al., 2021). Global studies have consistently shown that cultivating forage crops can ameliorate saline–alkali soils (Xia et al., 2019; Feng et al., 2023; Zhang et al., 2023). Long-term forage cultivation enhances soil nutrient availability via root exudation, thereby improving microbial microenvironments and influencing soil microbial dynamics (Cui et al., 2021; Momesso et al., 2022; Yan et al., 2022). Although microbial communities under forage crops such as *Medicago sativa* L., *Trifolium pratens*e L., *Elymus sibiricus* L., and *Leymus chinensis* have been extensively studied (Fu et al., 2021; Wahdan et al., 2021; Liu et al., 2022; Yan et al., 2022), the long-term effects of forage cultivation on microbial functional diversity remain less clear. In addition, differential patterns of root exudation among forage species may distinctly shape soil microbial community structure and function (Zhou et al., 2017). The rhizosphere, a unique microenvironment strongly shaped by root exudation, serves as a highly dynamic interface connecting soil, microorganisms, and plants (Hinsinger et al., 2009). As an integrated symbiotic system, it embodies complex interactions across organic, inorganic, biotic, and abiotic processes. Accordingly, numerous studies have examined the relationships between rhizosphere microbial diversity, environmental variables, and ecosystem functions (Jiang et al., 2022; Lu et al., 2022; Ren et al., 2022).

Alfalfa (*Medicago sativa* L.) is a high-quality leguminous forage and one of the most widely cultivated worldwide (Li and Brummer, 2012). Its symbiosis with nitrogen-fixing rhizobia makes it indispensable for crop rotation and the restoration of degraded lands (Jones et al., 2007). In the rhizobium–legume symbiosis, plants supply nutrients and energy to rhizobia for nitrogen fixation, which in turn influences plant growth and the broader soil microbial environment (Song et al., 2021). Rhizobial inoculation has been shown to increase activities of key enzymes, including superoxide dismutase (SOD), peroxidase (POD), and catalase (CAT) (Grover et al., 2011; Shrivastava and Kumar, 2015), and to enhance the production of organic acids such as oxalic, citric, succinic, and malic acids (Deakin and Broughton, 2009). Collectively, these findings highlight the potential for effective rhizosphere microorganisms to promote plant biomass and improve soil microenvironments, including saline–alkali soils. Thus, clarifying the spatiotemporal response mechanisms of alfalfa rhizosphere microorganisms in saline–alkali environments is vital for guiding soil improvement strategies. Despite this importance, it remains unclear whether soil enzyme activities in saline–alkali soils follow patterns similar to those of successional shifts in alfalfa rhizosphere microbial communities. To address this gap, we investigated the temporal dynamics of rhizosphere microbial communities in alfalfa across different soil types and evaluated the role of rhizosphere microorganisms in improving these environments. Using next-generation sequencing (NGS), complemented by soil enzyme assays and physicochemical analyzes, we characterized how rhizosphere bacterial communities respond to both temporal and spatial variation across three saline–alkali soils representing different alfalfa cultivation ages.

## 2 Materials and methods

### 2.1 Sample collection

A total of 36 rhizosphere and bulk soil samples were collected from *Medicago varia* Martin. cv. Caoyuan No. 3 across four cultivation ages (1, 3, 5, and 8 years) and three soil types. Four cultivation ages (1, 3, 5, and 8 years), with each year having three replicates, not different growth stages during each year. Sampling was done at the beginning of the flowering period of each growing year, with three replicates. Sampling occurred at three different locations: (1) Jigesitai Town, Dalat Banner, Ordos City, Inner Mongolia (hereafter Jigesitai; N40°33′96.10″, E110°51′22.10″; elevation 1100 m); soil pH = 8.20, (2) Shaerqin Township, Tumet Left Banner, Hohhot City, Inner Mongolia (hereafter Shaerqin; N40°34′54.33″, E111°46′52.11″; elevation 1050 m; soil pH = 8.8); and (3) Hailiu Village, Tumet Left Banner, Hohhot City, Inner Mongolia (hereafter Hailiu; N40°31′17″, E111°23′46.34″; elevation 1008 m; soil pH: 9.20). All sites are located within the saline–alkali improvement zone of the Yellow River Basin and experience a semi-arid continental monsoon climate, with mean annual temperatures of 6.1–7.3 °C, mean annual precipitation of 240–362 mm, and an average frost-free period of 135 days.

Soil samples (0–20 cm depth) were randomly collected from three healthy plants using a soil auger as a single sample. Loose soil was removed, and the root samples were immediately placed in 50 ml sterile centrifuge tubes and refrigerated. In the laboratory, roots with clay soil were rinsed twice with sterile water to collect rhizosphere soil. The washings were centrifuged at 4 °C (10,000 × g for 10 min), and the precipitate was the rhizosphere soil, stored at -20 °C for microbial assays. Under the three different soil conditions, full-length PacBio sequencing of rhizosphere soil microorganisms and Illumina sequencing of root alfalfa of the same variety at four different growth years were performed. Sample codes reflected site, growth age, and sample number. The first letter denotes the site (S = Shaerqin, J = Jigesitai, H = Hailiu), the second letter denotes cultivation age (O = 1 year, T = 3 years, F = 5 years, E = 8 years), and the third character “S” denotes soil. For example, HOS1 refers to the first rhizosphere soil sample collected from first-year alfalfa at Hailiu. Approximately 50 plants (n = 50) were excavated per sampling event. Loose soil was removed, and soil tightly adhering to roots was defined as rhizosphere soil. Plant residues and coarse fragments were eliminated using a 2 mm sieve.

A portion of each sample was flash-frozen in liquid nitrogen, packed on dry ice, and shipped to Beijing Biomarker Technologies Co., Ltd. (www.biomarker.com.cn) for microbial sequencing. The remaining portion was used to measure soil physicochemical properties and enzyme activities.

### 2.2 Determination of soil physicochemical properties

Rhizosphere soil nutrient content and physicochemical properties were determined following standard procedures. Soil pH was measured using a calibrated pH meter (PHS-3C) and standard buffer solutions. Soil organic matter was quantified in accordance with Chinese National Standard GB 9884–88. Mineral elements, total phosphorus, and total potassium were measured by atomic absorption spectrophotometry. Total organic carbon (TOC) and total nitrogen were determined using a total organic carbon analyzer. Each measurement was performed in triplicate.

### 2.3 Soil enzymology

To preserve enzyme activity, all analyzes were conducted on freshly collected rhizosphere soil. Enzyme activities were measured following the methods of Das et al (Callahan et al., 2016). Eleven enzymes were analyzed: (i) five carbon-cycle enzymes (α-glucosidase, β-glucosidase, β-xylosidase, β-N-acetylglucosaminidase, β-cellobiosidase); (ii) two nitrogen-cycle enzymes (urease, protease); (iii) three oxidoreductases—one reductase (dehydrogenase) and two oxidases (phenol oxidase, peroxidase); and (iv) one phosphorus-cycle enzyme (phosphatase).

### 2.4 DNA extraction and sequencing

Total genomic DNA (gDNA) was extracted from 0.25 g of each prepared soil sample using a QIAGEN DNeasy PowerSoil Kit (Mo Bio Laboratories, Solanan Beach, CA, USA) following the manufacturer’s protocol. DNA quality and quantity were assessed by the ratios of 260 nm/280 nm and 260 nm/230 nm. For full-length bacterial 16S rRNA gene amplification, we performed PCR for each sample using primer sets of 27F (5′-AGRGTTTGATYNTGGCTCAG -3′) and 1492R (5′-TASGGHTACCTTGTTASGACTT -3′) with adapter sequences and barcode sequences. The PCR was performed in a total reaction volume of 10µL: 2 × KOD FX NEo Buffer 5.0 µL, DNA template (100 ng/mL) 2.2 µL, prime1 (10 µM) 0.3 µL, prime2 (10 µM) 0.3 µL, dNTP 2.0 µL, KOD FX NEo 0.2 µL. After an initial denaturation at 95°C for 5 min, an amplification was performed by 30 cycles of incubations for 30 sec at 95°C, 30 sec at 50°C, and 6 sec at 72°C, followed by a final extension at 72°C for 7 min. Then the amplified products were purified and recovered using 1.0% agarose gel electrophoresis method. Finally, the library construction and sequencing steps were performed by Beijing Biomarker Technologies Co.Ltd, in which library preparing using SMRTbell Express Template Prep Kit 2.0.

The bioinformatic analysis in this study was completed at the Biomarker biocloud platform (www.biocloud.org). To obtain the raw tags, paired-end reads were merged by FLASH(v1.2.7, http://ccb.jhu.edu/software/FLASH/). Then raw tags were filtered and clustered in the next steps. The merged tags were compared to the primers, and the tags with more than six mismatches were discarded by FASTX-Toolkit. Tags with an average quality score <20 in a 50 bp sliding window were truncated using Trimmomatic (http://www.usadellab.org/cms/?page=trimmomatic) and tags shorter than 350 bp were removed. We identified possible chimeras by employing UCHIME, a tool included in mothur (http://drive5.com/uchime). The denoised sequences were clustered using USEARCH (version 10.0) and tags with similarity >=97% were regarded as a OTU. Taxonomy was assigned to all OTUs by searching against the Silva databases (Release132, http://www.arb-silva.de.) using the uclust within QIIME. Neighbor-Joining phylogenies (PhyNJ) were inferred with MEGAN5 (http://ab.inf.uni-tuebingen.de/software/megan5).

Alpha diversity indices referring to community diversity (Shannon), community richness (Chao1 and ACE) were calculated by Mothur (version 1.30 http://www.mothur.org). Beta diversity analysis was conducted using QIIME to analyze the distribution of the soil microbial community. Principal component analysis (PCA) was conducted to explore the differences between the microbial community structure of all soil samples. principal coordinate analysis (PcoA) was used to assess the overall variations of microbial community structure.

### 2.5 Sequence analysis

Bacterial diversity was assessed using the full-length 16S rRNA sequencing using PacBio platform. Paired-end sequencing libraries were constructed and processed following standard protocols (Callahan et al., 2016). After read merging and quality filtering, operational taxonomic units (OTUs) or amplicon sequence variants (ASVs) were generated through clustering or denoising, followed by taxonomic annotation and abundance profiling (Edgar et al., 2011). Subsequent analyzes included alpha and beta diversity, differential species abundance, correlation analysis, and functional prediction. Mitochondrial and chloroplast sequences were removed to avoid host contamination (Mcdonald et al., 2012). The OTU table was generated following the method of Edgar (2013). Sequencing depth fidelity was assessed using rarefaction curves (QIIME diversity alpha-rarefaction). For alpha and beta diversity analyzes, high-quality reads were clustered into OTUs, and Shannon indices and UniFrac distance matrices were calculated accordingly. Sequences were clustered at 97% similarity using USEARCH v10.0 (Lin et al., 2021), and OTUs representing <0.005% of total sequences were removed as recommended by (Bokulich et al., 2013; Edgar, 2013). Alpha diversity, reflecting species richness and diversity within samples, was estimated using the Chao1, ACE, Shannon, and Simpson indices (Meng et al., 2023). Beta diversity was analyzed in QIIME to evaluate community compositional similarity among samples. For beta diversity distance calculations, the binary Jaccard algorithm, which considers presence/absence of taxa, was used to generate pairwise beta distance values (Ferger et al., 2017).

### 2.6 Statistical analysis

Independent Student’s *t*-tests and Student–Newman–Keuls one-way ANOVA were performed in SPSS 25.0 (SPSS, Chicago, USA) to assess differences among groups representing distinct soil types (Wang et al., 2021a). Statistical significance was defined as *P* < 0.05. Principal coordinate analysis (PCoA) was carried out using the vegan package in R (version 3.6.2) (Xie et al., 2021) and visualized using ggplot2. Heatmaps were generated using the R heatmap package to display species abundance patterns and identify dominant taxa.

## 3 Results and discussion

### 3.1 Soil physicochemical properties across soil types

Grassland resistance and resilience are often evaluated within the framework of site-level water management. The physical and chemical properties of rhizosphere soils collected from alfalfa grown in different regions are summarized in **Table 1**. As expected, soil texture varied distinctly among the three soil types. All soils were alkaline, and pH values showed slight but consistent saline–alkali soils (8.20 to 9.20). This pattern suggests that land degradation may contribute to gradual acidification. Soil pH is widely regarded as a “master soil variable” because it governs key biological, chemical, and physical processes that regulate plant growth and biomass production (Hinsinger et al., 2009; Yan et al., 2015). Minor shifts in pH can therefore influence soil nutrient availability, organic matter turnover, enzyme activity, and stress tolerance. These subtle pH changes likely reflect interactions among alkaline earth metals, soil acid ions, mineral weathering, and microbial activity.

**Table 1:** Basic properties and nutrient characteristics of the three soil types.

Sampling locations	pH	Total nitrogen (g/kg)	Total phosphorus (g/kg)	Total potassium (g/kg)	Exchangeable calcium (g/kg)	Exchangeable magnesium (mg/kg)
Shaerqin (S)	8.80 ± 0.11a	2.07 ± 0.05a	1.30 ± 0.10a	14.00 ± 2.06a	7.56 ± 0.72a	35.22 ± 4.60a
Jigesitai (J)	8.20 ± 0.07a	2.13 ± 0.16a	1.38 ± 0.06a	14.20 ± 1.45a	7.47 ± 0.71a	33.24 ± 6.79a
Hailiu (H)	9.20 ± 0.23a	2.10 ± 0.15a	1.26 ± 0.05a	13.21 ± 0.65a	6.84 ± 0.35a	26.99 ± 0.73a
Sampling locations		Available manganese (μmol/g)	Available copper (μmol/g)	Available zinc (μmol/g)	TOC (g/kg)	T-AOC (U/mg)
Shaerqin (S)		0.54 ± 0.06a	4.82 ± 0.15a	1.12 ± 0.10a	11.12 ± 1.64a	8.96 ± 3.65a
Jigesitai (J)		0.57 ± 0.05a	4.57 ± 0.18a	1.16 ± 0.05a	9.88 ± 1.16a	8.36 ± 1.21a
Hailiu (H)		0.57 ± 0.04a	4.48 ± 0.19a	1.07 ± 0.06a	10.44 ± 2.05a	9.99 ± 0.96a

Different letters indicate significant differences among treatments at *P* < 0.05 based on Tukey’s HSD test (n = 3). Soil enzyme designation codes: Hailiu (H), Jigesitai (J), and Shaerqin (S).

Clay and silt contents were highest in loam soils, intermediate in sandy soils, and lowest in saline–alkali soils, consistent with expected textural differences. Exchangeable magnesium also varied significantly (**Table 1**), with markedly higher values in loam and sandy soils. This pattern may indicate a long history of phosphorus fertilization in these regions (Ibrahim et al., 2021; Liu et al., 2021). The reduced concentrations of alkaline earth metals in saline–alkali soils may partly explain their lower pH. Compared with unplanted soils, saline/sandy loam soils supporting alfalfa exhibited reduced exchangeable calcium, exchangeable magnesium, and total organic carbon, ranging from 6.84–7.56 mg/kg, 26.99–35.22 mg/kg, and 9.88–11.12 g/kg, respectively (**Table 1**).

Total nitrogen, total phosphorus, total potassium, available magnesium, available copper, and available zinc did not differ significantly among rhizosphere soils. Nonetheless, land degradation can profoundly alter soil physicochemical properties and, consequently, soil biological community structure. Shifts in nutrient pools—including available N, P, K, and organic matter—can disrupt soil homeostasis and influence a wide range of ecological processes (Lin et al., 2021). For example, heavy-metal inputs can trigger new chemical reactions, suppress microbial activities essential for nutrient cycling, and induce broader changes in soil physical and chemical characteristics (Jaiswal and Pandey, 2018).

### 3.2 Spatiotemporal variation in soil enzyme activities

**Figure 1:**
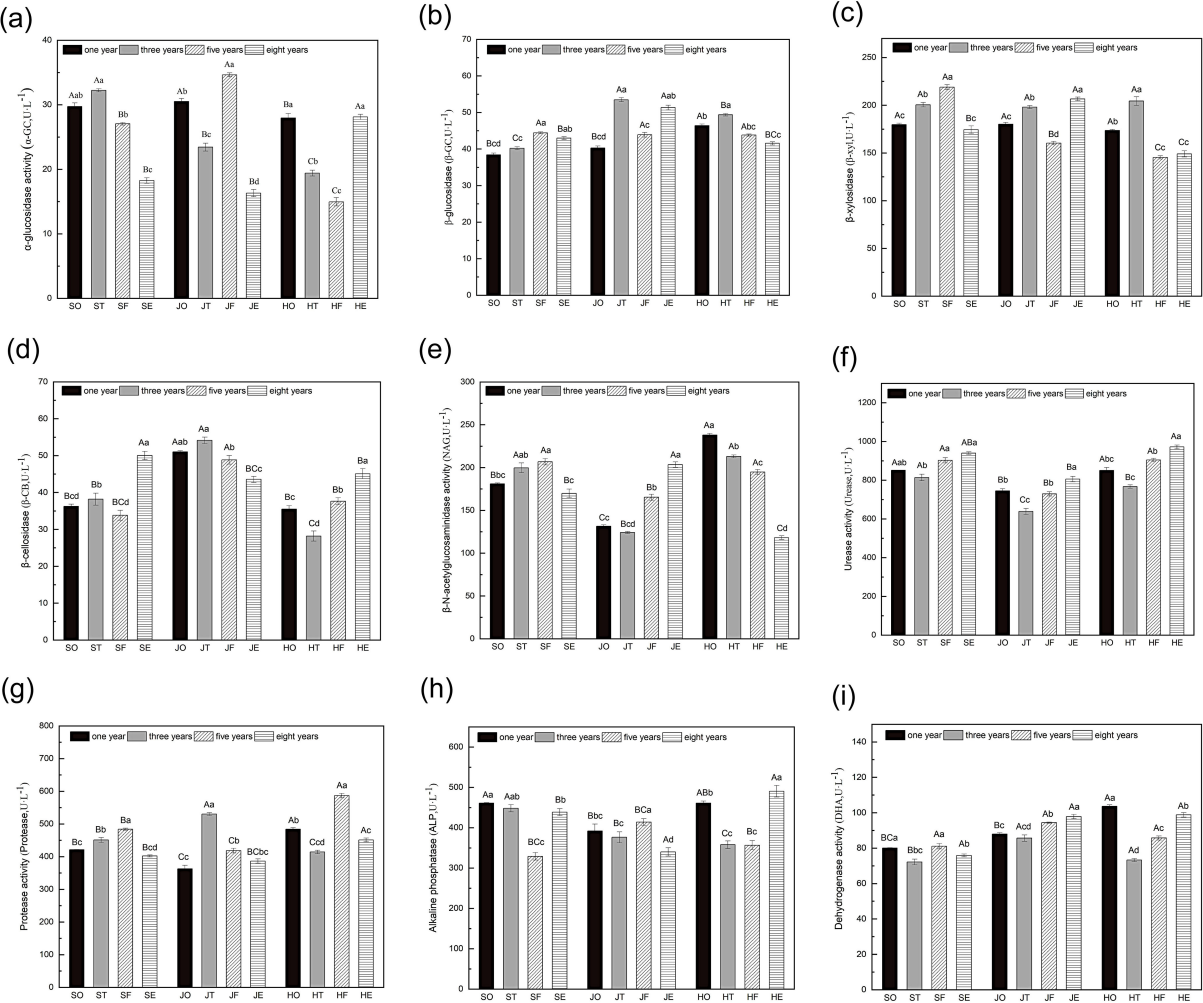
Soil enzyme activities in *Medicago varia* Martin. cv. Caoyuan No. 3 rhizosphere soil (n = 3). Soil enzyme analysis codes: Hailiu (H), Jigesitai (J), Shaerqin (S). **(a)** α-glucosidase activity; **(b)** β-glucosidase; **(c)** β-xylosidase; **(d)** β-cellosidase; **(e)** β-N-acetylglucosaminidase activity; **(f) **Urease activity; **(g)** Protease activity; **(h)** Alkaline phosphatase; **(i)** Dehydrogenase activity. Three samples were collected from each region at four cultivation ages.

Spatiotemporal shifts in rhizosphere enzyme activities associated with C/N cycling, oxidoreductases, and phosphatases are presented in **Figure 1**. Across regions, α-glucosidase activity generally declined with increasing alfalfa cultivation years (*P* < 0.001), except in Hailiu, where the HE samples showed an increase rather than a decrease (**Figure 1g**). β-Glucosidase exhibited the opposite pattern: activity increased over time in Shaerqin and Jigesitai but declined in Hailiu (**Figure 1a**). For β-cellobiosidase, enzyme activity in Shaerqin and Hailiu increased by 43.5% and 23.2%, respectively, after eight years of cultivation, whereas Jigesitai showed a 12.3% decline (**Figure 1b**). β-Xylosidase activity remained largely unchanged across all sites, suggesting that it does not function as a key enzyme in these soils (**Figure 1c**). β-N-Acetylglucosaminidase displayed region-specific patterns, likely reflecting differences in soil properties (**Figure 1d**).

Cellulose, hemicellulose, and lignin degradation capacities are widespread among soil bacteria such as *Bacillus*, *Cellulomonas*, and *Streptomyces (*Jiang et al., 2022; Lu et al., 2022), and cellulose hydrolysis contributes positively to soil structural and functional improvement (Wang et al., 2021; Ren et al., 2022). Consistent with this, Shaerqin soils showed enhanced hydrolytic capacity for cellulose (β-cellobiosidase, β-glucosidase) and hemicellulose (β-xylosidase, β-N-acetylglucosaminidase) with increasing cultivation age, indicating heightened metabolic activity at the root–soil interface. In contrast, Jigesitai exhibited markedly different trajectories, likely due to weaker plant growth, nutrient-poor sandy soils, and reduced microbial activity. Even so, several cellulose and hemicellulose hydrolases showed localized increases. Hailiu soils exhibited declining activity for most hydrolases, except β-cellobiosidase, suggesting weakening soil-improving effects under severe degradation. Because β-glucosidase activity is strongly dependent on soil organic matter content (Lin et al., 2021), these patterns collectively indicate that alfalfa’s ability to enhance soil quality diminishes as degradation intensifies.

For N-cycle enzymes, protease activity fluctuated during intermediate years but ultimately showed no significant net change compared with the first year (**Figure 1e**). Urease activity increased steadily across all regions (**Figure 1f**). Because urease catalyzes the hydrolysis of urea to ammonia and carbon dioxide, its increase indicates enhanced N transformation and utilization with longer alfalfa cultivation (Yang et al., 2018). Dehydrogenase activity, alkaline phosphatase was elevated in both sandy and saline–alkali soils relative to lightly degraded soils (**Figure 1**). Phosphatase activity did not differ significantly among regions. High salinity is known to induce oxidative stress, often resulting in elevated oxidase activity (Alzate Zuluaga et al., 2021). Overall, C/N-cycling enzymes and oxidoreductases displayed strong spatial and temporal variation, reflecting inherent differences in C and N cycling across soil types (Ibrahim et al., 2021; Saghaï et al., 2022) and indicating oxidative stress under varying degrees of degradation (Zeng et al., 2021).

### 3.3 Diversity of the microbial community

#### 3.3.1 Alpha diversity

**Figure 2:**
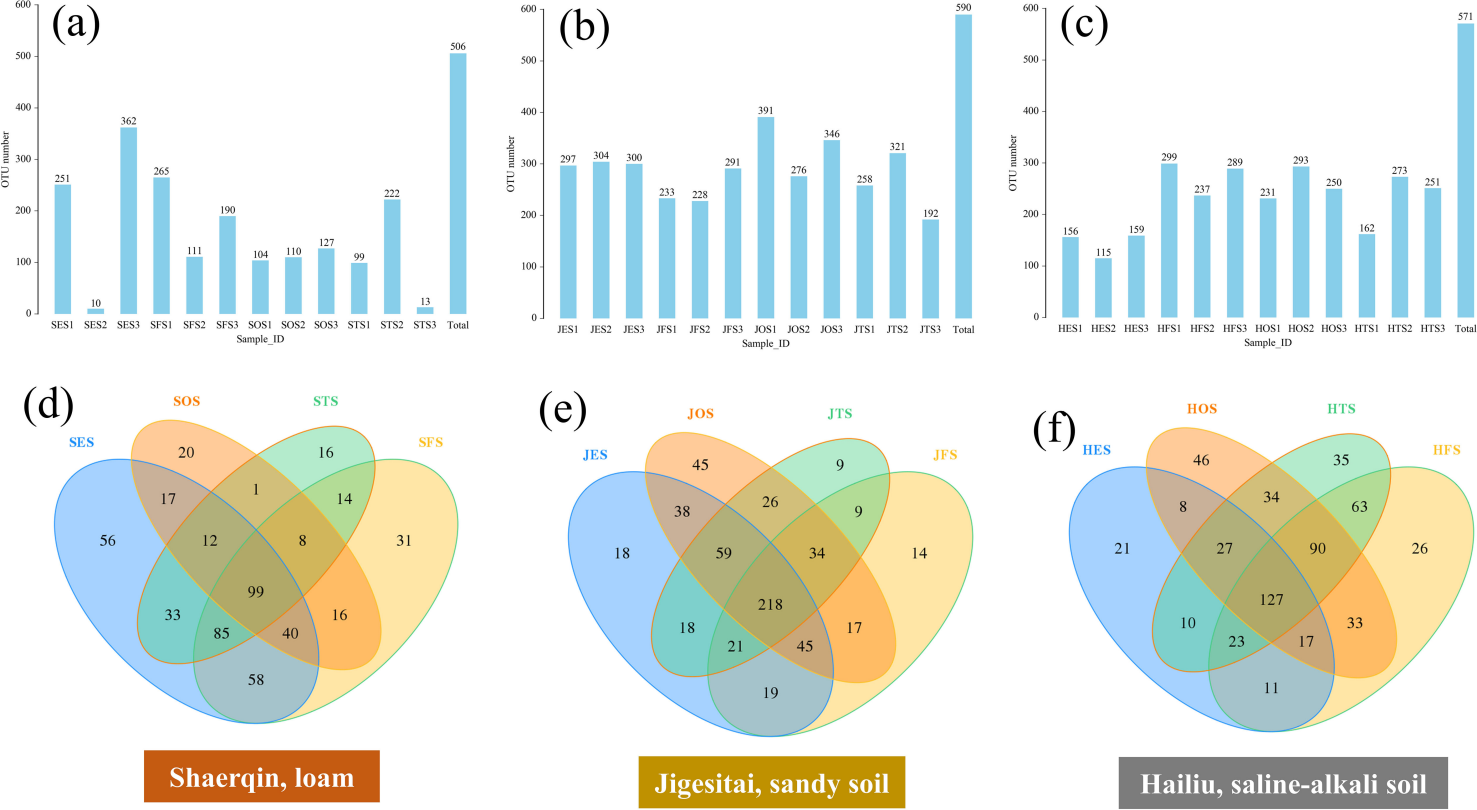
Species number diagrams **(a–c)** and Venn diagrams **(d–f)**. Soil enzyme analysis codes: Hailiu (H), Jigesitai (J), Shaerqin (S). Three soil samples were collected at four cultivation ages: one year (O), two years (T), five years (F), and eight years (E).

Alpha diversity was used to assess the composition and temporal dynamics of rhizosphere-associated bacterial communities across the three regions. Overall, the detected numbers of OTUs followed the pattern Jigesitai (mild saline-alkali soil), Hailiu (heavy saline-alkali soil) and Shaerqin (moderate saline-alkali soil). This ranking may partly reflect two anomalous Shaerqin samples (SES2 and STS3). In the Shaerqin loam region, OTU numbers declined progressively with increasing alfalfa cultivation years, likely due to nutrient depletion and a consequent reduction in bacterial populations under long-term monoculture (**Figure 2a**). Jigesitai sandy soils showed a slight but consistent decline in OTUs (**Figure 2b**), consistent with previous observations (Xia et al., 2019). In contrast, the Hailiu salt–alkali soils exhibited a clear upward trend in OTU abundance (**Figure 2c**), which may reflect gradual improvements in rhizosphere microenvironmental conditions during prolonged alfalfa cultivation.

**Figure 3:**
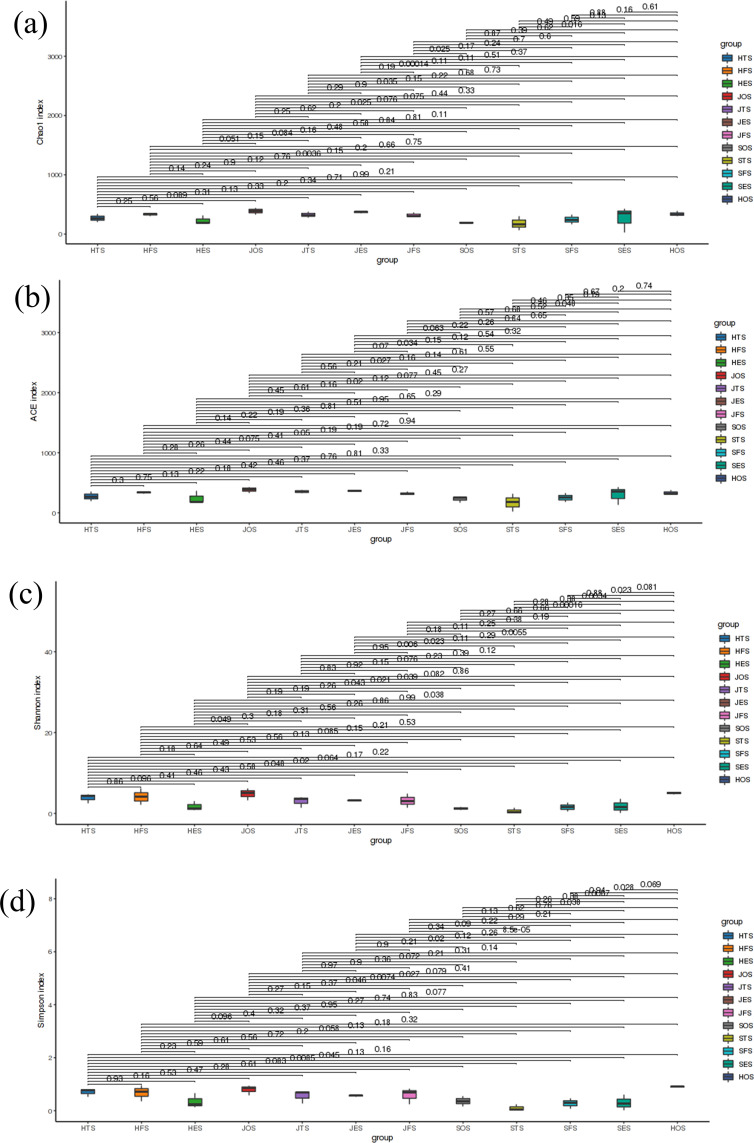
α-Diversity patterns show that microbial diversity declined along the soil degradation gradient. **(a)** Chao1 index; **(b)** ACE index; **(c)** Shannon index; (ad) Simpson index.

OTU overlap among growth years also declined with extended cultivation. For example, in Shaerqin, overlap between the first-year (SOS) samples and later years declined markedly—STS (120/268), SFS (163/351), and SES (168/400)—indicating progressive divergence in community structure with long-term planting (**Figure 3d**). Similar patterns were observed in Jigesitai and Hailiu soils (**Figures 2e**, **3f**).

Together, these findings indicate strong temporal dependence of rhizosphere microbial community composition during alfalfa growth (Zhang et al., 2023). To further characterize the rhizosphere microbiome, we quantified species richness (Chao1, ACE) and diversity (Shannon, Simpson) for all 36 samples (**Figure 3**). These measures revealed a clear diversity gradient across degraded land types. At one year of growth, microbial diversity followed the pattern Jigesitai sandy soil > Hailiu salt–alkali soil > Shaerqin loam soil. With increasing cultivation years, diversity declined in both the sandy and salt–alkali soils, but increased in the loam soils of Shaerqin. This contrasting pattern may reflect stronger sensitivity of sandy-soil microbial symbioses to continuous cropping, where long-term monoculture reduces network complexity (Xia et al., 2019). In loam soils, a higher organic matter content likely supports greater functional diversity and resilience (Zhang et al., 2023).

In saline–alkali soils, limited metabolic substrate availability likely drove the observed decline in diversity over time, highlighting the importance of organic matter inputs for sustaining microbial communities (Hinsinger et al., 2009; Chen et al., 2021). Decreases in cellulose- and hemicellulose-hydrolyzing enzyme activities further support this explanation. Because hydrolysates of cellulose and hemicellulose are key substrates for microbial metabolism and essential for microbial growth and survival (Yan et al., 2022; Feng et al., 2023), reduced enzyme activity would constrain microbial turnover. Indeed, trends in hydrolase activities paralleled those of microbial diversity, underscoring the influence of microbial diversity on soil organic matter (SOM) conversion processes (Momesso et al., 2022).

#### 3.3.2 Beta diversity

**Figure 4:**
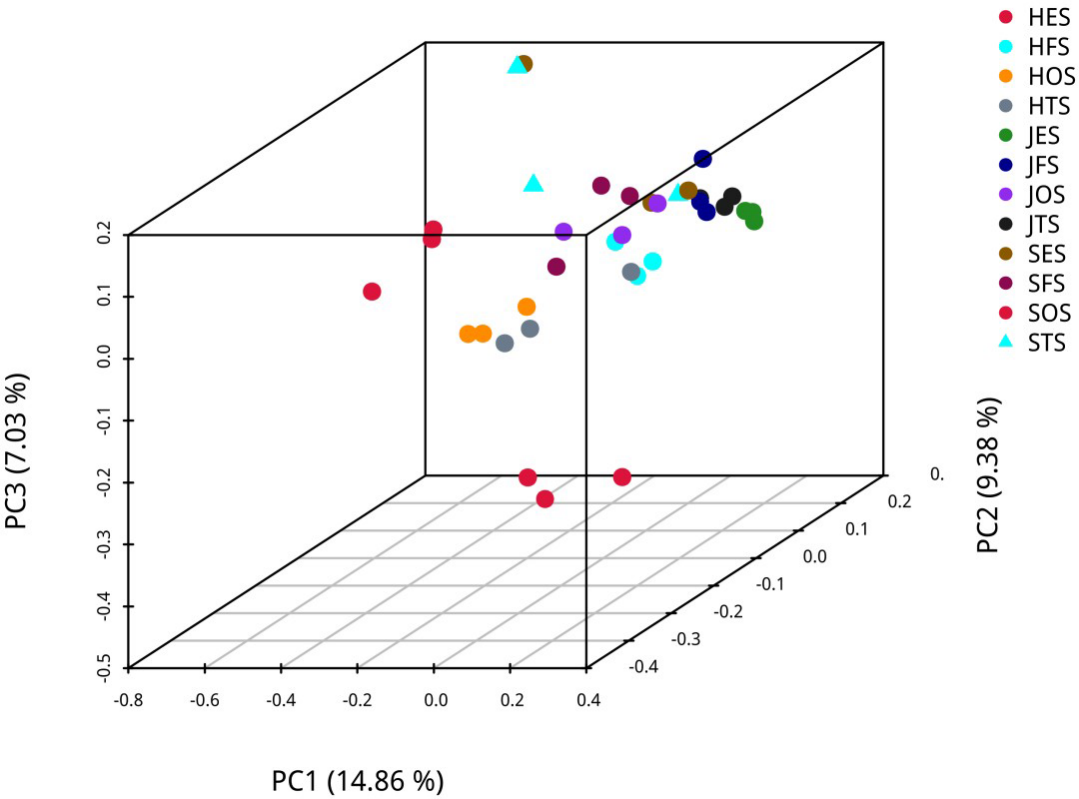
PCoA measured by weighted/unweighted UniFrac shows that the largest separation between microbial communities is the sampling place (PC1), followed by growth year (PC2), and intergroup differences (PC3).

To identify the major factors shaping community structure among samples, we assessed β diversity using principal coordinate analysis (PCoA) across all datasets (**Figure 4**). Overall, the first two axes explained 24.24% of the total variance. PC1 (14.86%) primarily separated samples by niche, whereas PC2 (9.38%) reflected variation associated with alfalfa growth year. Rhizosphere soil and root samples occupied distinct regions of the ordination space, and the 36 samples clustered into 12 discrete groups, further demonstrating that the rhizosphere bacterial communities differed markedly from the endophytic communities.

In Jigesitai and Shaerqin, samples showed progressively tighter clustering with increasing alfalfa growth years, suggesting that microbial community composition in sandy and loam soils gradually stabilized over time. In contrast, samples from the Hailiu saline–alkali soil increasingly diverged with longer cultivation, indicating that microbial compositional differences intensified and that community structures continued to undergo directional succession. Across all degraded land types, bacterial community abundance declined gradually with the natural succession associated with increasing alfalfa growth years. This pattern may reflect the well-established inhibitory effects of salinized soils on rhizosphere microbial diversity and abundance (Wang et al., 2021b). Sandy loam is considered the most suitable soil type for alfalfa cultivation, which is consistent with its comparatively stable microbial community structure (Rocheleau et al., 2011). Our findings indicate that planting alfalfa in suitable soil promotes stabilization of rhizosphere microbial communities over time, and that a stable microbial structure enhances plant disease resistance and stress tolerance, as well as the resilience of the rhizosphere microenvironment (Cui et al., 2021; Fu et al., 2021).

### 3.4 Bacterial communities

**Figure 5:**
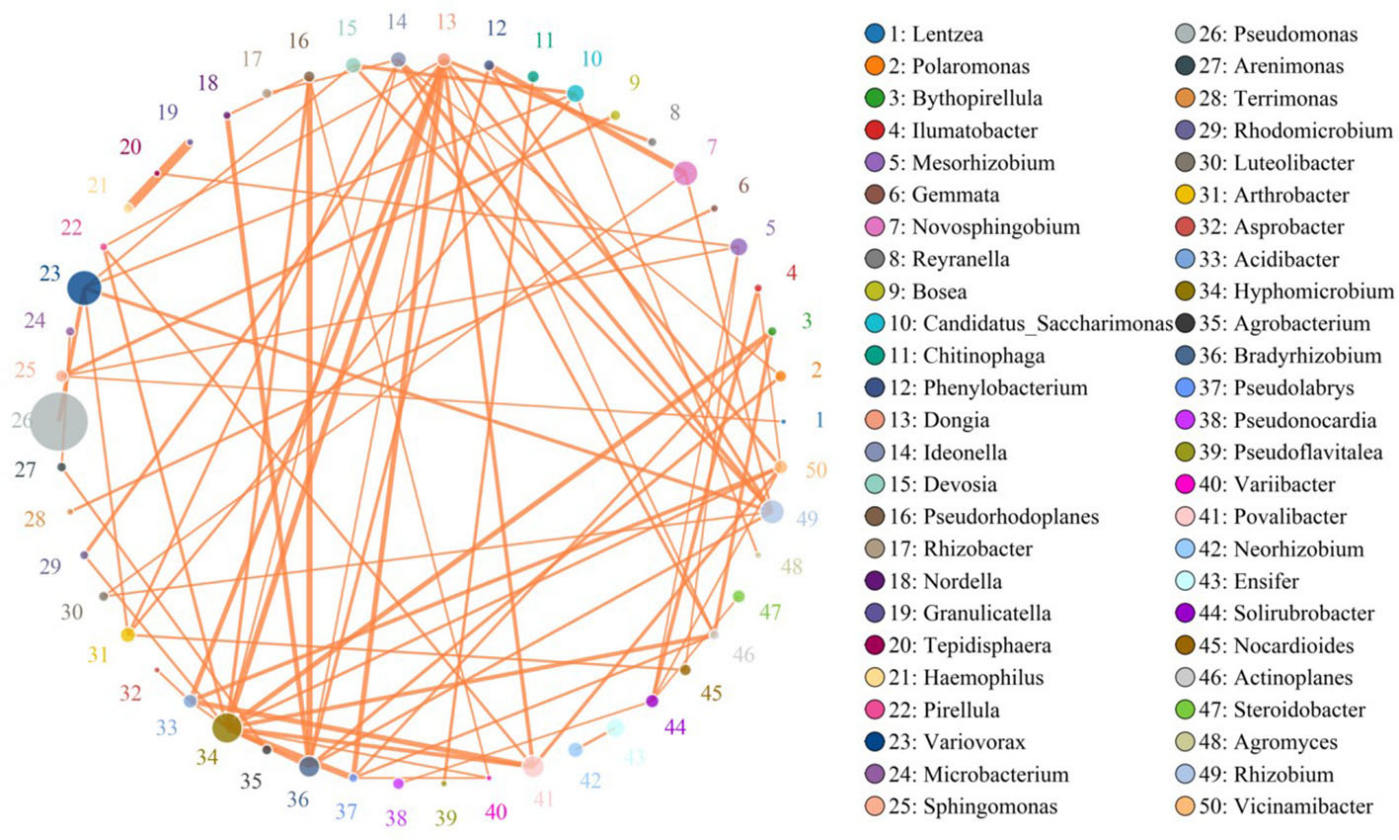
Neural network plot showing the microbial community composition of all rhizosphere samples.

High-throughput sequencing revealed that microbial community composition in the alfalfa root environment shifted substantially in response to soil degradation. As shown in **Figure 5**, a total of 50 genera were detected, spanning 10 phyla and accounting for more than 90% of all high-quality sequence tags. *Pseudomonas*, *Variovorax*, *Hyphomicrobium*, *Rhizobium*, *Novosphingobium*, *Solirubrobacter*, and *Bradyrhizobium* were consistently dominant. In SE samples, *Limnohabitans* represented the major taxon, with a relative abundance of 5.5–8.7%, compared with only 0.9–2.1% in HE samples (**Figure 5b**). Notably, *Akkermansia* emerged as a dominant genus (22.1–27.6%) in HE samples. Overall, soil degradation in alfalfa-growing regions shifted rhizosphere microbial dominance from *Bacillus*-associated taxa toward *Akkermansia*.

**Figure 6:**
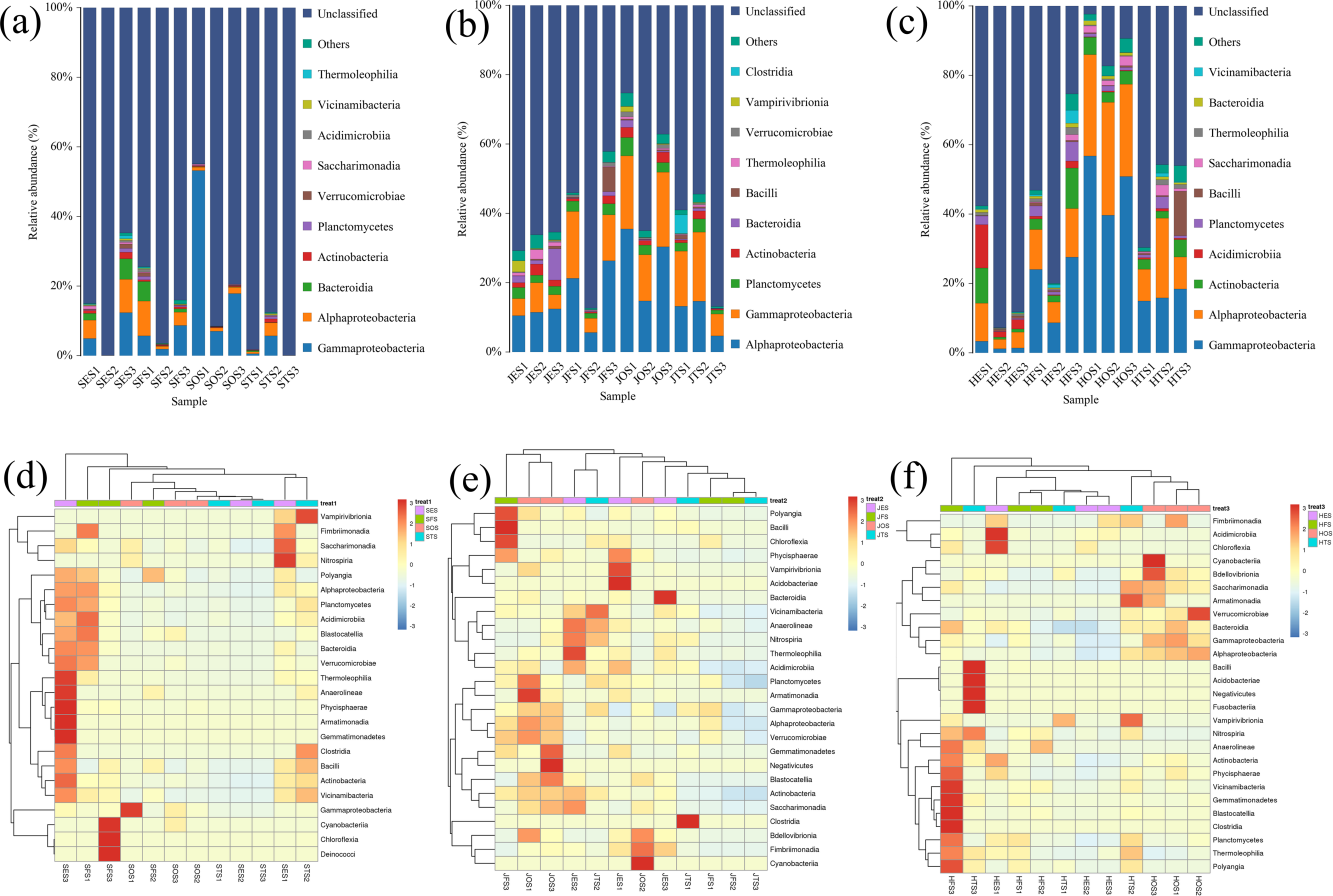
**(a–c)** The relative abundance of major rhizosphere bacterial classes in Hailiu (H), Jigesitai (J), and Shaerqin (S), respectively. **(d–f)** Heatmaps of rhizosphere bacterial abundance at the class level for Hailiu (H), Jigesitai (J), and Shaerqin (S).

Class-level profiles further illustrated how rhizosphere bacterial communities changed with alfalfa growth years (**Figures 6a–c**). Gammaproteobacteria and Alphaproteobacteria were the most abundant classes across all soils. In first-year plants, Proteobacteria levels were highest in Hailiu, intermediate in Jigesitai, and lowest in Shaerqin. With increasing cultivation years, Shaerqin showed marked enrichment of Bacteroidia, whereas Jigesitai exhibited increased proportions of Bacilli, Planctomycetes, Actinobacteria, and Bacteroidia. Hailiu similarly accumulated Planctomycetes, Acidimicrobiia, Actinobacteria, and Bacteroidia. A hierarchical clustering heatmap at the genus level (**Figure 6b**) demonstrated that samples of similar planting years grouped together, indicating that plant age—interacting with soil properties and enzyme activities (**Figures 6d–f**; **Table 2**; **Figure 1**)—was a major driver of rhizosphere microbial composition.

**Table 2:** PCR computer test procedure.

Steps	Temperature	Time	Cycles
Predegeneration	98 °C	45 s	1
Denaturation	98 °C	10 s	30
Annealing	55-65 °C	20-30s	
Delay	72 °C	10–30 s/kb	
Terminal extension	72 °C	1–5 min	1
Hold	4-12 °C	25-35	1

Phylogenetic clustering revealed no strong hierarchical separation among dominant taxa (left dendrograms in **Figures 6d–f**), with most major groups co-occurring alongside less abundant species, reflecting a shared ecological niche. Dominant gram-negative groups included Bacteroidia and Alpha/Gammaproteobacteria, both characteristic of eutrophic environments (Wahdan et al., 2021; Liu et al., 2022). Their prevalence in Shaerqin aligns with its comparatively fertile soil. By contrast, gram-positive *Bacilli* and *Clostridia*, capable of forming long-lived endospores under unfavorable conditions such as drought or temperature extremes (Zhou et al., 2017), were more prominent in the nutrient-poor soils of Jigesitai and Hailiu. The detection of *Acinetobacter*, a WHO-listed “priority pathogen” with high antibiotic resistance, in sandy and saline-alkali soils indicates that potential pathogens also inhabit alfalfa rhizospheres in degraded environments. Collectively, rhizosphere dominance patterns diversified with increased planting years and varied substantially across soils of different degradation levels.

The relationship between biodiversity and ecosystem multifunctionality depends strongly on environmental context (Hu et al., 2021). Loss of plant and rhizosphere microbial diversity is particularly detrimental under severe drought conditions, underscoring the need for climate-specific conservation strategies (Worlanyo and Jiangfeng, 2021). Consistent with earlier findings (Chen and Chen, 2021b; Cao et al., 2022; Mei et al., 2022), our results showed that degraded soils differed markedly in physicochemical properties and enzyme activities, and these parameters changed systematically with alfalfa growth age. Nutrient dynamics were closely linked to soil enzymatic profiles, jointly shaping rhizosphere microbial community assembly. The positive association between rhizosphere microbial multifunctionality and drought gradients observed here aligns with previous reports (Jing et al., 2015; Delgado-Baquerizo et al., 2016). Overall, alfalfa cultivation in degraded soils improved soil physicochemical properties, enhanced enzyme activity, and enriched the structure and diversity of rhizosphere microbial communities. These findings provide new insights into the spatiotemporal dynamics of rhizosphere microbes in forage crops grown under degraded soil conditions. Future research should integrate multi-omics, long-term trials, and diverse environments to elucidate mechanisms and enhance soil restoration strategies.

## 5 Conclusion

This study demonstrates a clear association between rhizosphere microecological characteristics and the degree of soil degradation. Although degraded soils alter both the composition and abundance of rhizosphere microorganisms, mild degradation can be partially mitigated through continuous alfalfa cultivation, which reshapes microbial community structure and reduces the negative effects of suboptimal soil conditions. Our findings indicate that long-term alfalfa cultivation regulates rhizosphere health by modifying nutrient availability, decreasing peroxidase activity, and enriching beneficial microbial taxa. Rhizosphere microbial diversity declined with increasing planting years in saline-alkali and sandy soils but increased in loam soil. Moreover, prolonged cultivation promoted greater stability of the microbial community in sandy and loam soils, whereas the microbial assemblages in saline-alkali soil became increasingly dispersed and heterogeneous. Overall, these results suggest that sustained alfalfa cultivation contributes to the ecological recovery of degraded soils and that a stable rhizosphere microbial community is a key mechanism supporting soil restoration. Key limitations include restricted soil types, limited functional validation, and short temporal scale. Future research should integrate multi-omics, long-term trials, and diverse environments to elucidate mechanisms and enhance soil restoration strategies.

## Data Availability

The original contributions presented in the study are included in the article/supplementary material. Further inquiries can be directed to the corresponding authors.

## References

[B1] Alzate Zuluaga M. Y. Milani K. M. L. Miras-Moreno M. B. Lucini L. Valentinuzzi F. Mimmo T. . (2021). The adaptive metabolomic profile and functional activity of tomato rhizosphere are revealed upon PGPB inoculation under saline stress. Environ. Exp. Bot. 189, 104552. doi: 10.1016/j.envexpbot.2021.104552. PMID: 38826717

[B2] Bokulich N. A. Subramanian S. Faith J. J. Gevers D. Gordon J. I. Knight R. . (2013). Quality-filtering vastly improves diversity estimates from Illumina amplicon sequencing. Nat. Methods 10, 57–59. doi: 10.1038/nmeth.2276. PMID: 23202435 PMC3531572

[B3] Callahan B. J. McMurdie P. J. Rosen M. J. Han A. W. Johnson A. J. A. Holmes S. P. (2016). DADA2: high-resolution sample inference from Illumina amplicon data. Nat. Methods 13, 581–583. doi: 10.1038/nmeth.3869. PMID: 27214047 PMC4927377

[B4] Cao T. Fang Y. Chen Y. Kong X. Yang J. Alharbi H. . (2022). Synergy of saprotrophs with mycorrhiza for litter decomposition and hotspot formation depends on nutrient availability in the rhizosphere. Geoderma 410, 115662. doi: 10.1016/j.geoderma.2021.115662. PMID: 38826717

[B5] Chen X. Chen H. Y. H. (2021b). Plant mixture balances terrestrial ecosystem C:N:P stoichiometry. Nat. Commun. 12, 4562. doi: 10.1038/s41467-021-24889-w. PMID: 34315908 PMC8316448

[B6] Chen X. Jiang C. Zheng L. Zhang L. Fu X. Chen S. . (2021). Evaluating the genesis and dominant processes of groundwater salinization by using hydrochemistry and multiple isotopes in a mining city. Environ. pollut. 283, 117381. doi: 10.1016/j.envpol.2021.117381. PMID: 34034018

[B7] Cui J. Song D. Dai X. Xu X. He P. Wang X. . (2021). Effects of long-term cropping regimes on SOC stability, soil microbial community and enzyme activities in the Mollisol region of Northeast China. Appl. Soil Ecol. 164, 103941. doi: 10.1016/j.apsoil.2021.103941. PMID: 38826717

[B8] Deakin W. J. Broughton W. J. (2009). Symbiotic use of pathogenic strategies: rhizobial protein secretion systems. Nat. Rev. Microbiol. 7, 312–320. doi: 10.1038/nrmicro2091. PMID: 19270720

[B9] Delgado-Baquerizo M. Maestre F. T. Reich P. B. Jeffries T. C. Gaitan J. J. Encinar D. . (2016). Microbial diversity drives multifunctionality in terrestrial ecosystems. Nat. Commun. 7, 10541. doi: 10.1038/ncomms10541. PMID: 26817514 PMC4738359

[B10] Edgar R. C. (2013). UPARSE: highly accurate OTU sequences from microbial amplicon reads. Nat. Methods 10, 996–998. doi: 10.1038/nmeth.2604. PMID: 23955772

[B11] Edgar R. Haas B. Clemente J. Quince C. Knight R. (2011). UCHIIME improves sensitivity and speed of chimera detection. Bioinf. (Oxford England) 27, 2194–2200. doi: 10.1093/bioinformatics/btr381. PMID: 21700674 PMC3150044

[B12] Feng Y. Xu X. Liu J. Han J. Lu H. (2023). Planting Suaeda salsa improved the soil properties and bacterial community diversity in a coastal mudflat. Land Degrad. Dev. 34, 3262–3271. doi: 10.1002/ldr.4681. PMID: 41531421

[B13] Ferger S. W. Peters M. K. Appelhans T. Detsch F. Hemp A. Nauss T. . (2017). Synergistic effects of climate and land use on avian beta-diversity. Divers. Distrib. 23, 1246–1255. doi: 10.1111/ddi.12615. PMID: 40046247

[B14] Fu B. Li Z. Gao X. Wu L. Lan J. Peng W. (2021). Effects of subsurface drip irrigation on alfalfa (Medicago sativa L.) growth and soil microbial community structures in arid and semi-arid areas of northern China. Appl. Soil Ecol. 159, 103859. doi: 10.1016/j.apsoil.2020.103859. PMID: 38826717

[B15] Grover M. Ali S. Z. Sandhya V. Rasul A. Venkateswarlu B. (2011). Role of microorganisms in adaptation of agriculture crops to abiotic stresses. World J. Microbiol. Biotechnol. 27, 1231–1240. doi: 10.1007/s11274-010-0572-7. PMID: 30311153

[B16] Heng T. He X. Yang L. Xu X. Feng Y. (2021). Mechanism of saline-alkali land improvement using subsurface pipe and vertical well drainage measures and its response to agricultural soil ecosystem. Environ. pollut., 118583. doi: 10.1016/j.envpol.2021.118583. PMID: 34861335

[B17] Hinsinger P. Bengough A. G. Vetterlein D. Young I. M. (2009). Rhizosphere: biophysics, biogeochemistry and ecological relevance. Plant Soil 321, 117–152. doi: 10.1007/s11104-008-9885-9. PMID: 30311153

[B18] Hu W. Ran J. Dong L. Du Q. Ji M. Shuran Y. . (2021). Aridity-driven shift in biodiversity–soil multifunctionality relationships. Nat. Commun. 12, 5350. doi: 10.1038/s41467-021-25641-0. PMID: 34504089 PMC8429721

[B19] Ibrahim M. M. Zhang H. Guo L. Chen Y. Heiling M. Zhou B. . (2021). Biochar interaction with chemical fertilizer regulates soil organic carbon mineralization and the abundance of key C-cycling-related bacteria in rhizosphere soil. Eur. J. Soil Biol. 106, 103350. doi: 10.1016/j.ejsobi.2021.103350. PMID: 38826717

[B20] Jaiswal D. Pandey J. (2018). Impact of heavy metal on activity of some microbial enzymes in the riverbed sediments: ecotoxicological implications in the Ganga River (India). Ecotox. Environ. Safe. 150, 104–115. doi: 10.1016/j.ecoenv.2017.12.015. PMID: 29272714

[B21] Jiang Q. Lu W. Zhang L. Jin Y. Wang Y. Chen J. . (2022). Promotion mechanism of self-transmissible degradative plasmid transfer in maize rhizosphere and its application in naphthalene degradation in soil. J. Environ. Sci. 115, 240–252. doi: 10.1016/j.jes.2021.07.014. PMID: 34969451

[B22] Jing X. Sanders N. J. Shi Y. Chu H. Classen A. T. Zhao K. . (2015). The links between ecosystem multifunctionality and above- and belowground biodiversity are mediated by climate. Nat. Commun. 6, 8159. doi: 10.1038/ncomms9159. PMID: 26328906 PMC4569729

[B23] Jones K. M. Kobayashi H. Davies B. W. Taga M. E. Walker G. C. (2007). How rhizobial symbionts invade plants: the Sinorhizobium–Medicago model. Nat. Rev. Microbiol. 5, 619–633. doi: 10.1038/nrmicro1705. PMID: 17632573 PMC2766523

[B24] Li X. Brummer E. C. (2012). Applied genetics and genomics in alfalfa breeding. Agronomy 2, 40–61. doi: 10.3390/agronomy2010040. PMID: 30654563

[B25] Lin H. Liu C. Li B. Dong Y. (2021). Trifolium repens L. regulated phytoremediation of heavy metal contaminated soil by promoting soil enzyme activities and beneficial rhizosphere associated microorganisms. J. Hazard. Mater. 402, 123829. doi: 10.1016/j.jhazmat.2020.123829. PMID: 33254810

[B26] Liu B. Hu Y. Wang Y. Xue H. Li Z. Li M. (2022). Effects of saline-alkali stress on bacterial and fungal community diversity in Leymus chinensis rhizosphere soil. Environ. Sci. pollut. Res. 29, 70000–70013. doi: 10.1007/s11356-022-20270-6. PMID: 35579830

[B27] Liu Y. You C. Zhang Y. Chen S. Zhang Z. Li J. . (2021). Resistance and resilience of grasslands to drought detected by SIF in inner Mongolia, China. Agr. For. Meteorol., 308–309, 108567. doi: 10.1016/j.agrformet.2021.108567. PMID: 38826717

[B28] Lu S. He Y. Chen Y. Chen L. Wang Z. Yuan J. . (2022). Co-analysis of rhizosphere metabolomics and bacterial community structures to unfold soil ecosystem health in Camellia oleifera land under long-term cultivation. Appl. Soil Ecol. 171, 104336. doi: 10.1016/j.apsoil.2021.104336. PMID: 38826717

[B29] Mcdonald D. Clemente J. Kuczynski J. Rideout J. R. Stombaugh J. Doug W. . (2012). The Biological Observation Matrix (BIOM) format or: how I learned to stop worrying and love the ome-ome. GigaScience 1, 7. doi: 10.1186/2047-217X-1-7. PMID: 23587224 PMC3626512

[B30] Mei L. Zhang P. Cui G. Yang X. Zhang T. Guo J. (2022). Arbuscular mycorrhizal fungi promote litter decomposition and alleviate nutrient limitations of soil microbes under warming and nitrogen application. Appl. Soil Ecol. 171, 104318. doi: 10.1016/j.apsoil.2021.104318. PMID: 38826717

[B31] Meng F. Harkes P. van Steenbrugge J. J. M. Geissen V. (2023). Effects of microplastics on common bean rhizosphere bacterial communities. Appl. Soil Ecol. 181, 104649. doi: 10.1016/j.apsoil.2022.104649. PMID: 38826717

[B32] Momesso L. Crusciol C. A. C. Leite M. F. A. Bossolani J. W. Kuramae E. E. (2022). Forage grasses steer soil nitrogen processes, microbial populations, and microbiome composition in a long-term tropical agriculture system. Agric. Ecosyst. Environ. 323, 107688. doi: 10.1016/j.agee.2021.107688. PMID: 38826717

[B33] Ren X. Wang L. Tang J. Sun H. Giesy J. P. (2022). Combined effects of degradable film fragments and micro/nanoplastics on growth of wheat seedling and rhizosphere microbes. Environ. pollut. 294, 118516. doi: 10.1016/j.envpol.2021.118516. PMID: 34864099

[B34] Rocheleau S. Kuperman R. G. Dodard S. G. Sarrazin M. Savard K. Paquet L. . (2011). Phytotoxicity and uptake of nitroglycerin in a natural sandy loam soil. Sci. Total Environ. 409, 5284–5291. doi: 10.1016/j.scitotenv.2011.08.067. PMID: 21975007

[B35] Saghaï A. Wittorf L. Philippot L. Hallin S. (2022). Loss in soil microbial diversity constrains microbiome selection and alters the abundance of N-cycling guilds in barley rhizosphere. Appl. Soil Ecol. 169, 104224. doi: 10.1016/j.apsoil.2021.104224. PMID: 38826717

[B36] Schmid M. W. van Moorsel S. J. Hahl T. De Luca E. De Deyn G. B. Wagg C. . (2021). Effects of plant community history, soil legacy and plant diversity on soil microbial communities. J. Ecol. 109, 3007–3023. doi: 10.1111/1365-2745.13714. PMID: 40046247

[B37] Sherif M. I. Sultan M. Sturchio N. C. (2019). Chlorine isotopes as tracers of solute origin and age of groundwaters from the Eastern Desert of Egypt. Earth Planet. Sci. Lett. 510, 37–44. doi: 10.1016/j.epsl.2018.12.035. PMID: 38826717

[B38] Shrivastava P. Kumar R. (2015). Soil salinity: a serious environmental issue and plant growth promoting bacteria as one of the tools for its alleviation. Saudi J. Biol. Sci. 22, 123–131. doi: 10.1016/j.sjbs.2014.12.001. PMID: 25737642 PMC4336437

[B39] Song T. Sun N. Dong L. Cai H. (2021). Enhanced alkali tolerance of rhizobia-inoculated alfalfa correlates with altered proteins and metabolic processes as well as decreased oxidative damage. Plant Physiol. Biochem. 159, 301–311. doi: 10.1016/j.plaphy.2020.12.021. PMID: 33418189

[B40] Wahdan S. F. M. Heintz-Buschart A. Sansupa C. Tanunchai B. Wu Y. T. Schädler M. . (2021). Targeting the active rhizosphere microbiome of Trifolium pratense in grassland evidences a stronger-than-expected belowground biodiversity-ecosystem functioning link. Front. Microbiol. 12, 629169. doi: 10.3389/fmicb.2021.629169. PMID: 33597941 PMC7882529

[B41] Wang S. Sun L. Narsing Rao M. P. Wang L. Wang Y. Li W.-J. (2021). “ Insights into the microbial diversity in saline-alkaline soils of China,” in Microbial Communities and their Interactions in the Extreme Environment. Eds. Egamberdieva D. Birkeland N.-K. Li W.-J. Panosyan H. ( Springer Singapore, Singapore), 17–41.

[B42] Wang Y. Wang M. Chen Y. Li C. Zhou Z. (2021a). Microbial community structure and co-occurrence are essential for methanogenesis and its contribution to phenanthrene degradation in paddy soil. J. Hazard. Mater. 417, 126086. doi: 10.1016/j.jhazmat.2021.126086. PMID: 34020358

[B43] Wang Z. Zhu Y. Li N. Liu H. Zheng H. Wang W. . (2021b). High-throughput sequencing-based analysis of the composition and diversity of endophytic bacterial community in seeds of saline-alkali tolerant rice. Microbiol. Res. 250, 126794. doi: 10.1016/j.micres.2021.126794. PMID: 34062342

[B44] Worlanyo A. S. Jiangfeng L. (2021). Evaluating the environmental and economic impact of mining for post-mined land restoration and land-use: a review. J. Environ. Manage. 279, 111623. doi: 10.1016/j.jenvman.2020.111623. PMID: 33223352

[B45] Xia J. Ren J. Zhang S. Wang Y. Fang Y. (2019). Forest and grass composite patterns improve the soil quality in the coastal saline-alkali land of the Yellow River Delta, China. Geoderma 349, 25–35. doi: 10.1016/j.geoderma.2019.04.032. PMID: 38826717

[B46] Xie G. Kong X. Kang J. Su N. Luo G. Fei J. (2021). Community-level dormancy potential regulates bacterial beta-diversity succession during the co-composting of manure and crop residues. Sci. Total Environ. 772, 145506. doi: 10.1016/j.scitotenv.2021.145506. PMID: 33571759

[B47] Yan H. Gu S. Li S. Shen W. Zhou X. Yu H. . (2022). Grass-legume mixtures enhance forage production via the bacterial community. Agric. Ecosyst. Environ. 338, 108087. doi: 10.1016/j.agee.2022.108087. PMID: 38826717

[B48] Yan N. Marschner P. Cao W. Zuo C. Qin W. (2015). Influence of salinity and water content on soil microorganisms. Int. Soil Water Conserv. Res. 3, 316–323. doi: 10.1016/j.iswcr.2015.11.003. PMID: 38826717

[B49] Yang W. Jiao Y. Yang M. Wen H. (2018). Methane uptake by saline–alkaline soils with varying electrical conductivity in the Hetao Irrigation District of Inner Mongolia, China. Nutr. Cycling Agroecosyst. 112, 265–276. doi: 10.1007/s10705-018-9943-5. PMID: 30311153

[B50] Zeng H. Xu H. Liu G. Wei Y. Zhang J. Shi H. (2021). Physiological and metagenomic strategies uncover the rhizosphere bacterial microbiome succession underlying three common environmental stresses in cassava. J. Hazard. Mater. 411, 125143. doi: 10.1016/j.jhazmat.2021.125143. PMID: 33858103

[B51] Zhang G. Bai J. Zhai Y. Jia J. Zhao Q. Wang W. . (2023). Microbial diversity and functions in saline soils: A review from a biogeochemical perspective. J. Adv. Res. doi: 10.1016/j.jare.2023.06.015. PMID: 37392974 PMC11081963

[B52] Zhang Y. Dong S. Gao Q. Liu S. Ganjurjav H. Wang X. . (2017). Soil bacterial and fungal diversity differently correlated with soil biochemistry in alpine grassland ecosystems in response to environmental changes. Sci. Rep. 7, 43077. doi: 10.1038/srep43077. PMID: 28262753 PMC5338028

[B53] Zhou Y. Zhu H. Fu S. Yao Q. (2017). Variation in soil microbial community structure associated with different legume species is greater than that associated with different grass species. Front. Microbiol. 8. doi: 10.3389/fmicb.2017.01007. PMID: 28620371 PMC5449475

